# Transcriptomic Analysis of Fiber Strength in Upland Cotton Chromosome Introgression Lines Carrying Different *Gossypium barbadense* Chromosomal Segments

**DOI:** 10.1371/journal.pone.0094642

**Published:** 2014-04-24

**Authors:** Lei Fang, Ruiping Tian, Jiedan Chen, Sen Wang, Xinghe Li, Peng Wang, Tianzhen Zhang

**Affiliations:** National Key Laboratory of Crop Genetics and Germplasm Enhancement, Cotton Hybrid R & D Engineering Center (the Ministry of Education), Nanjing Agricultural University, Nanjing, China; New Mexico State University, United States of America

## Abstract

Fiber strength is the key trait that determines fiber quality in cotton, and it is closely related to secondary cell wall synthesis. To understand the mechanism underlying fiber strength, we compared fiber transcriptomes from different *G. barbadense* chromosome introgression lines (CSILs) that had higher fiber strengths than their recipient, *G. hirsutum* acc. TM-1. A total of 18,288 differentially expressed genes (DEGs) were detected between CSIL-35431 and CSIL-31010, two CSILs with stronger fiber and TM-1 during secondary cell wall synthesis. Functional classification and enrichment analysis revealed that these DEGs were enriched for secondary cell wall biogenesis, glucuronoxylan biosynthesis, cellulose biosynthesis, sugar-mediated signaling pathways, and fatty acid biosynthesis. Pathway analysis showed that these DEGs participated in starch and sucrose metabolism (328 genes), glycolysis/gluconeogenesis (122 genes), phenylpropanoid biosynthesis (101 genes), and oxidative phosphorylation (87 genes), etc. Moreover, the expression of MYB- and NAC-type transcription factor genes were also dramatically different between the CSILs and TM-1. Being different to those of CSIL-31134, CSIL-35431 and CSIL-31010, there were many genes for fatty acid degradation and biosynthesis, and also for carbohydrate metabolism that were down-regulated in CSIL-35368. Metabolic pathway analysis in the CSILs showed that different pathways were changed, and some changes at the same developmental stage in some pathways. Our results extended our understanding that carbonhydrate metabolic pathway and secondary cell wall biosynthesis can affect the fiber strength and suggested more genes and/or pathways be related to complex fiber strength formation process.

## Introduction

The cotton fiber is a terminally differentiated single cell derived from the epidermal cell of the developing ovule. After initiation, the fiber cell undergoes 1000- to 3000-fold elongation during its development. The development of cotton fibers involves four partially overlapping stages: initiation (−3 to +3 days post-anthesis; DPA), elongation and primary cell wall formation (3–23 DPA), secondary cell wall formation (16–40 DPA) and maturation (40–50 DPA) [1]–[6]. The most rapid period of fiber cell elongation begins around 10–16 DPA and continues to ∼20 DPA. Primary and secondary cell wall synthesis overlaps during the period of 16–25 DPA. During the secondary cell wall formation stage, the speed of cell elongation slows down and even stops.

Fiber strength is an important indicator of cotton fiber quality, and depends on formation of the secondary cell wall. Cellulose synthesis plays a predominant role in fiber cells, and cellulose accounts for >95% of the dry weight of the mature cotton fiber [3], [7]. Genome and EST sequencing have revealed that there are at least ten different CesA genes for cellulose synthase in *Arabidopsis*; CesA-like genes have also been reported in rice and barley [8]–[10]. In cotton (*Gossypium raimondii*), at least 15 cellulose synthase (CESA) sequences are required for cellulose synthesis [11]. A recent investigation in *Arabidopsis thaliana* using microarrays led to the identification of genes that are highly co-expressed with cellulose synthase genes and two mutants, irx8 and irx13, that have irregular xylem phenotypes, were also identified [12]. Sucrose synthase (Susy) is the enzyme that catalyzes the hydrolysis of sucrose to UDP-glucose that is then used as a substrate for cellulose synthesis. In cotton, the expression of Susy is higher at 16–32 DPA, and this enzyme plays a major role in partitioning carbon toward cellulose synthesis in the fiber [13]. SusC is another new sucrose synthase gene with a high level of expression during secondary cell wall synthesis [14]. Peroxide, mainly as H_2_O_2_, promotes cellulose synthesis as a signal of secondary cell wall synthesis [15], [16].

At present, many ovule- and fiber-specific cDNA libraries have been constructed and sequenced, and more than 268,000 expressed sequence tags (ESTs) from *Gossypium* are deposited in the NCBI database (http://www.ncbi.nlm.nih.gov). For genetic characterization of rapid cell elongation in cotton fibers, approximately 14,000 unique genes were assembled from 46,603 expressed sequence tags (ESTs) from developmentally-staged fiber cDNAs of a cultivated diploid species (*G. arboreum* L.). Eighty-one genes that were significantly up-regulated during secondary cell wall synthesis were found to be involved in cell wall biogenesis and energy/carbohydrate metabolism, which is consistent with the stage of cellulose synthesis during secondary cell wall modification in developing fibers [17]. Transcriptome profiling of the cotton fiber early in development by high-throughput tag-sequencing (Tag-seq) analysis using the Solexa Genome Analyzer reveals significant differential expression of genes in a fuzzless/lintless mutant [18]. High-throughput, genome-wide transcriptomic analysis of cotton under drought stress revealed a significant down-regulation of genes and pathways involved in fiber elongation, and an up-regulation of defense response genes [19]. More research have been processed in fiber initiation and elongation stage [20]–[24]. Saturated very-long-chain fatty acids (VLCFAs; C20:0–C30:0) exogenously applied in ovule culture medium significantly promoted fiber cell elongation in cotton (*G. hirsutum L.*) by activating ethylene biosynthesis [25], [26]. Previous investigations into cotton fiber development mainly focused on the elongation stage, and the number of genes reported from the later stages is quite small. Most of the genes up-regulated during secondary cell wall synthesis were related to cellulose synthesis, cell wall biosynthesis, and carbohydrate metabolism [17], [22], [27].

Chromosome segment introgression lines (CSILs) consist of a battery of near-isogenic lines that have been developed to cover the entire genomes of some crops, including tomato, rice, wheat, and cotton [28]–[31]. With the exception of a single, homozygous chromosome segment transferred from a donor parent, the remaining genome of each CSIL is the same as the recipient parent [31]. We used *G. barbadense* CSILs in the background of the standard genetic line of *G. hirsutum*, cv. TM-1, in order to understand the molecular mechanism behind superior quality fiber formation. Multi-point tests showed that three CSILs produced stronger fibers when compared to the recipient parent TM-1, but one CSIL produced weaker fibers. Using Solexa Genome sequencing, we analyzed transcriptome profiles from the CSILs and TM-1. We found that many genes were either up- or down-regulated at the stage of secondary cell wall synthesis, and that many metabolic pathways were altered in the CSILs.

## Materials and Methods

### Plant materials

*G. hirsutum* cv. TM-1, the genetic standard for Upland cotton, was obtained from the Southern Plains Agricultural Research Center, USDA-ARS, College Station/Texas, USA [32]. *G. barbadense* cv. Hai7124, an extra-long staple cotton that is widely grown in China, is descended from a selected individual in a study of inheritance of resistance to *Verticillium dahlia* [33], [34]. In this study, we identified three CSILs with stronger fiber or high fiber strength that carried different *G. barbadense* chromosome segment(s) in the recurrent parent TM-1. The detailed method of developing CSILs has been described previously [31]. We selected three CSILs, CSIL-35431, CSIL-31134, and CSIL-31010, in which the average fiber strength were 35.1, 34.73 and 34.28 cN/tex, respectively, significantly higher than TM-1, and also CSIL-35368 which had poorer fiber strength than TM-1(28.71 cN/tex) (Table S1). The introgressed *G. barbadense* chromosomal segments were different in the four lines [35]. Fiber samples were collected at 15, 20, and 25 DPA, frozen in liquid nitrogen, and stored at −70°C.

### RNA isolation and evaluation

Total RNA was extracted from frozen tissue using an improved CTAB extraction protocol [36]. RNAs were evaluated for quality using RNA Pico Chips on an Agilent 2100 Bioanalyzer (Agilent Technologies, Santa Clara, CA, USA). All RNA samples were quantified and qualified with an RNA Integrity Number (RIN) >8, and 28S/18S rRNA band intensity (2∶1).

### Library construction and sequencing

Digital gene expression libraries were constructed using the Illumina Gene Expression Sample Preparation Kit according to the manufacturer's instructions. We constructed and sequenced 14 libraries derived from immature fibers at 15, 20, and 25 DPA using the Solexa Genome Sequencing Analyzer system provided by BGI (Beijing Genomics Institute at Shenzhen, China), which gave 21 bp tags. The process was described in detail previously [18].

### Data processing, statistical evaluation, and selection of differentially expressed genes (DEGs)

Raw data reads were filtered by the Illumina pipeline to produce clean data. All low-quality data, such as short tags (<21 nt) and singletons, were removed. A database of 21-base-long sequences was produced beginning with CATG using 37,505 reference genes from the diploid species *G. raimondii* (http://www.phytozome.net). The remaining high quality sequences were then mapped to this database; only a single mismatch was allowed, and more than one match was excluded. Gene expression levels were the summation of tags aligned to the different positions of the same gene. Expression levels are expressed as TPM, transcripts per million. To identify DEGs during fiber elongation, we compared pairs of DEG profiles from different libraries. Three fiber development periods for the four CSILs were compared with the same period for TM-1, and 11 comparisons were obtained. P- and Q-values were also calculated for every comparison [37]. DEGs were defined as FDR≤0.001 with an absolute value of |log_2_Ratio|≥1 to judge the significance of differences in transcript abundance.

### Digital tag profiling analysis

DEG clustering in CSILs at different developmental stages were performed with Cluster3.0 (http://bonsai.hgc.jp/~mdehoon/software/cluster/software.htm). We also performed clustering with the ‘Self-organizing tree algorithm’ (SOTA, Multiple Array Viewer software, MeV 4.9.0) [38].

GO enrichment and KEGG (Kyoto Encyclopedia of Genes and Genomes) pathway analysis was done using BLAST2GO (http://www.blast2go.com/b2ghome). Mapman was also used to analyze metabolic pathway base on KEGG database [39].

### Quantitative RT-PCR

Quantitative RT-PCR assays were performed on a 7500 Real-Time PCR system (Applied Biosystems, San Francisco, CA, USA). Reactions were performed in a final volume of 15 µL and contained 2 µL of diluted cDNA, 7.5 µL of 2× SYBR mix (Roche, Basel, Switzerland), and 200 nM of the forward and reverse primers. Primer lengths were designed to be between 18 and 24 nt using Beacon Designer 7, and PCR amplicon lengths were designed to be between 100 bp and 150 bp (Table S2). The thermal cycling conditions were 40 cycles of 95°C for 15 s, 60°C for 30 s, and 72°C for 30 s. All reactions were run in triplicate, and the cotton *histone3* gene (ACC NO. AF024716) was used as an internal control for normalization of expression levels (F: 5′-GGTGGTGTGAAGAAGCCTCAT-3′, and R: 5′-AATTTCACGAACAAGCCTCTGGAA-3′). The relative gene expression levels were presented as 2^−ΔCT^.

## Results

### Statistical analysis of transcriptome data

The total number of sequence tags per library ranged from 7.0 to 8.5 million, and the number of distinct sequence tags was between 1.8 and 2.2 million. Approximately 50% of the clean tags were mapped to reference genes, and 60% of the reference genes were mapped with unambiguous tag (Table 1 and Table S3).

**Table 1:** The distribution of total and distinct tags.

Summary	TM-1			CSIL-35431			CSIL-31010		
	15DPA	20DPA	25DPA	15DPA	20DPA	25DPA	15DPA	20DPA	25DPA
Raw Data	8374304	7231305	14267931	7461562	7389820	7298224	7471212	7007447	7203054
Distinct Raw Tag	389162	394430	494345	375999	371748	317147	283130	337525	386302
Clean Tag	8144920	7013147	14002534	7264426	7213791	7124575	7369012	6840101	7002766
Distinct Clean Tag	169700	177887	251173	182629	199257	148121	182184	172775	188639
Unique Clean Tag Mapping to Gene	3983215	3206328	6829327	3961228	3411249	3463314	3671014	3381955	3552566
Total % of clean tag	48.90%	45.72%	48.77%	54.53%	47.29%	48.61%	49.82%	49.44%	50.73%
Unique Distinct Clean Tag Mapping to Gene	45380	40475	56172	53187	48453	41065	46720	45762	48147
Total % of distinct tag	26.74%	22.75%	22.36%	29.12%	24.32%	27.72%	25.64%	26.49%	25.52%
Unambiguous Tag-mapped Genes	21498	20901	22594	21781	22325	20590	21900	22457	22811
Percentage of reference genes	57.32%	55.73%	60.24%	58.07%	59.53%	54.90%	58.39%	59.88%	60.82%

Clean tags: tags after filtering dirty tags (low quality tags) from raw data.

Distinct tags: different kinds of tags.

Unambiguous tags: the clean tags after removing tags mapped to reference sequences from multiple genes.

**Figure 1:**
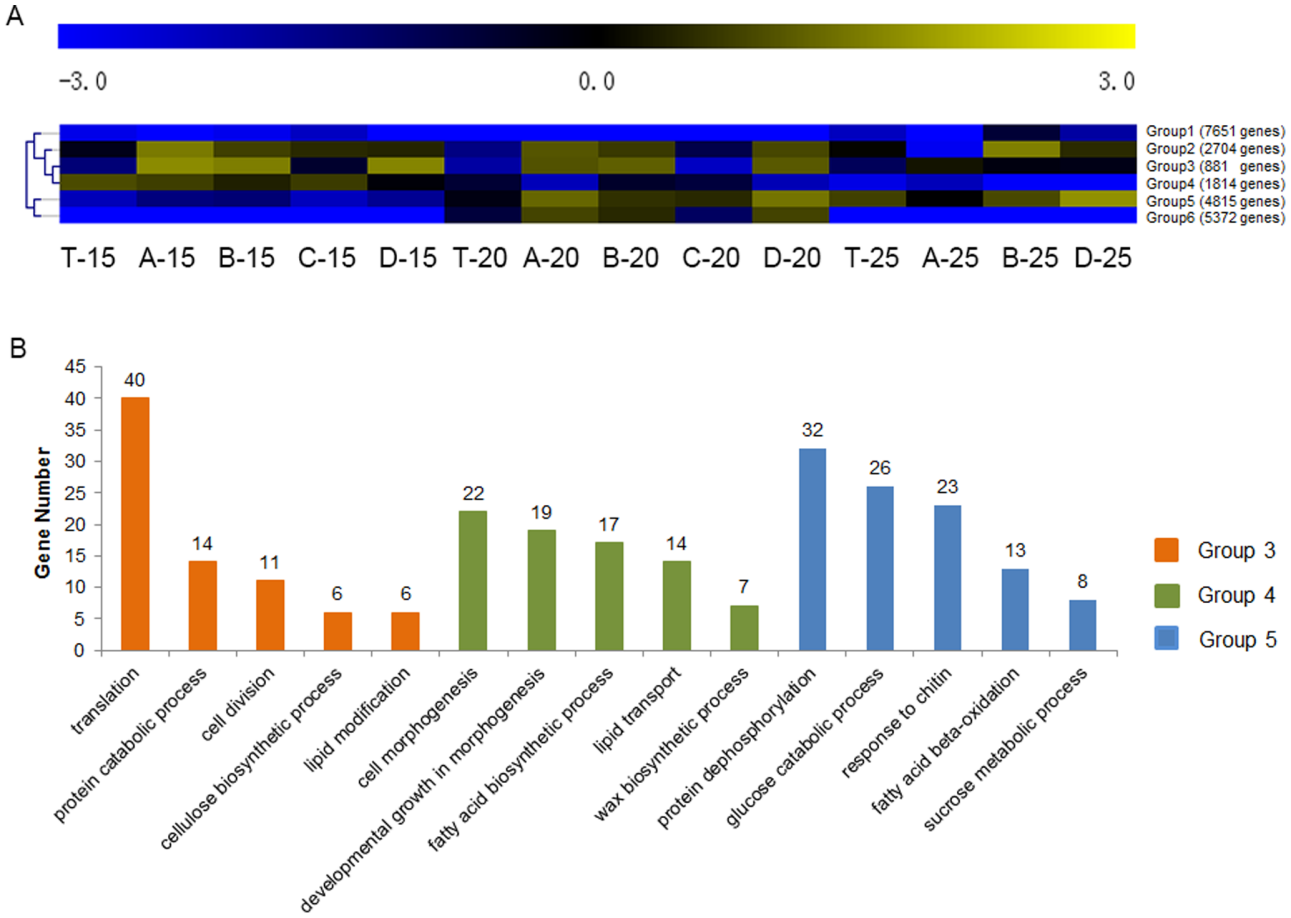
Statistical analysis of transcriptome data. (A) SOTA clustering of the different genes using Log2(TPM). T, TM-1; A, CSIL-35431; B, CSIL-31010; C, CSIL-31134; D, CSIL-35368. 15, 15 DPA; 20, 20 DPA; 25, 25 DPA. (B) Distribution of functions of genes in different clusters. Yellow square indicated group 3, green square indicated group 4 and blue square indicated group 5. X-axis indicated different enriched process and Y-axis indicated number of hit-found genes in these processes.

To see whether the fiber transcriptomes at different developmental stages were different, the 23,237 genes which were expressed in at least three libraries at one stage (15 DPA, 20 DPA, or 25 DPA) were classified into six groups using the Multiple Array Viewer using TPM value (Figure 1A). Genes in Group 3 had higher expression levels at 15 DPA and 20 DPA than at the later stage (25 DPA). Genes in Group 4 had higher expression levels at 15 DPA than at either 20 DPA or 25 DPA. Genes in Group 5 showed the opposite expression pattern, with higher expression levels at 20 DPA and 25 DPA compared to 15 DPA. The other groups also showed distinct expression patterns (Figure 1A).

Classification by gene function revealed that Group 3 is enriched in genes involved in protein catabolism, cell division, and cellulose biosynthesis, Group 4 is enriched in genes for cell morphogenesis, fatty acid biosynthesis, lipid transport, and wax biosynthesis, and Group 5 has more genes involved in glucose catabolism, response to chitin, and sucrose metabolism (Figure 1B). The unbalanced pattern of the expressed-gene functional distribution could possibly reflect some physiological events involved in secondary cell wall biosynthesis.

### Cluster analysis of differentially expressed genes (DEGs) between and/or among CSILs

**Figure 2:**
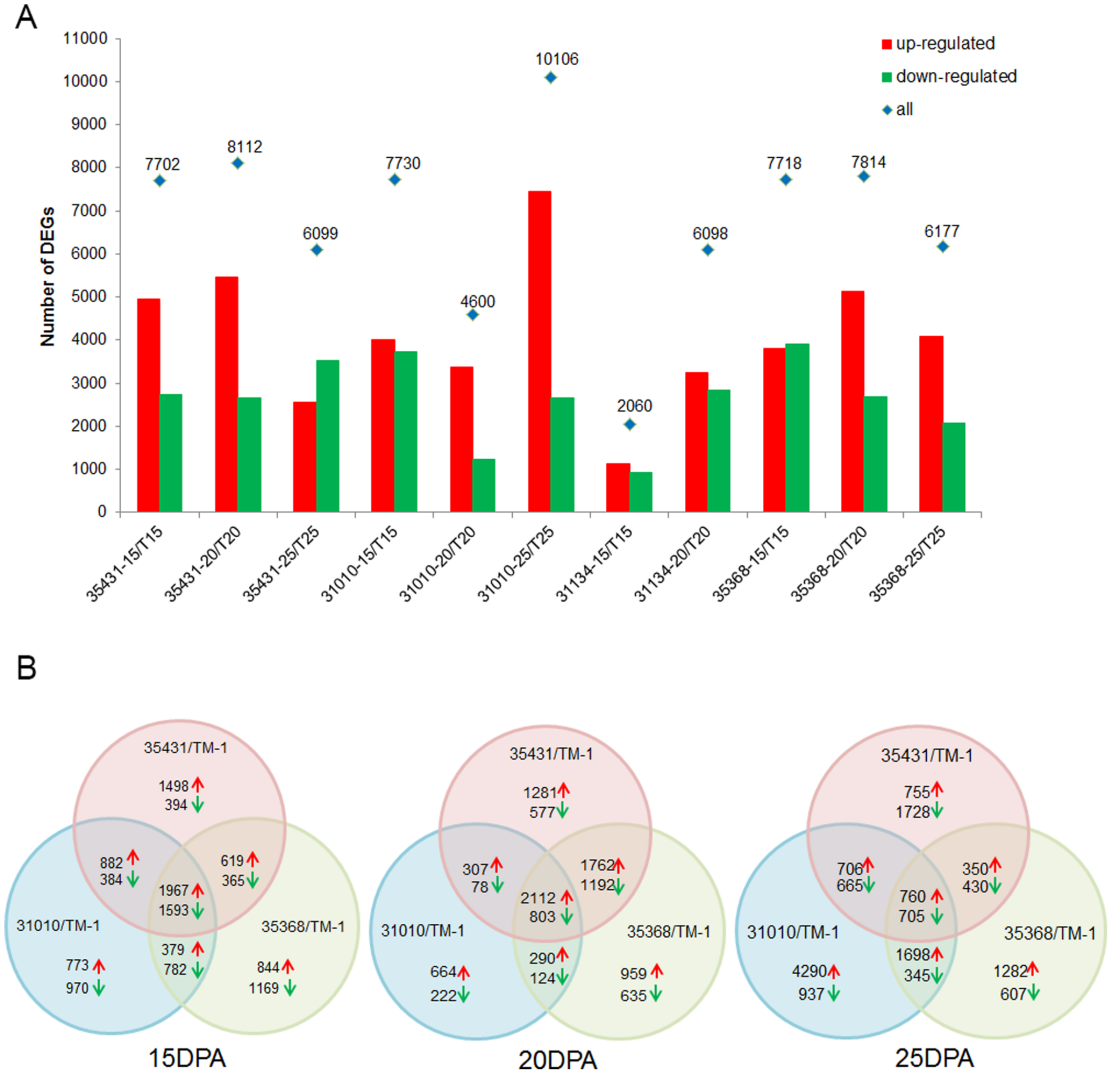
Statistical of DEGs between CSILs and TM-1 at 15, 20 and 25 DPA. (A) Up-regulated and down-regulated genes in different comparison. Red bar, up-regulated genes compared to TM-1; green bar, stand for down-regulated genes compared to TM-1, Blue square, total DEGs. CSILs included CSIL-35431, CSIL-3010, CSIL-31134, CSIL-35368 and TM-1. 15, 15DPA; 20, 20DPA; 25, 25DPA. (B) Common and special DEGs at 15 DPA, 20 DPA and 25 DPA.

We specifically looked for DEGs in secondary cell wall fibers from 15 to 25 DPA, because previous studies have reported that the different sets of transcripts responsible for fiber secondary cell wall formation may be enriched at these stages of development [17], [22], [27]. Three fiber development periods for the four CSILs were compared with TM-1 at the same period. DEGs were defined as FDR≤0.001 with an absolute value of |log_2_Ratio|≥1. Analysis of the data indicated that many genes showed differential expression in the 11 comparison groups. The number of DEGs were about 6,000–8,000 in CSILs from 15 DPA to 25 DPA (Figure 2A). But the number of DEGs in CSIL-31010 at 20 DPA, CSIL-31010 at 25 DPA, and CSIL-31134 at 15 DPA, were 4,600, 10,106 and 2,060, respectively. We also found that the DEGs that were up-regulated or down-regulated were different in CSILs. There were ∼1,500–3,500 DEGs in common from 15 DPA to 25 DPA between CSIL-35431, CSIL-31010, and CSIL-35368 (Figure 2B).

**Figure 3:**
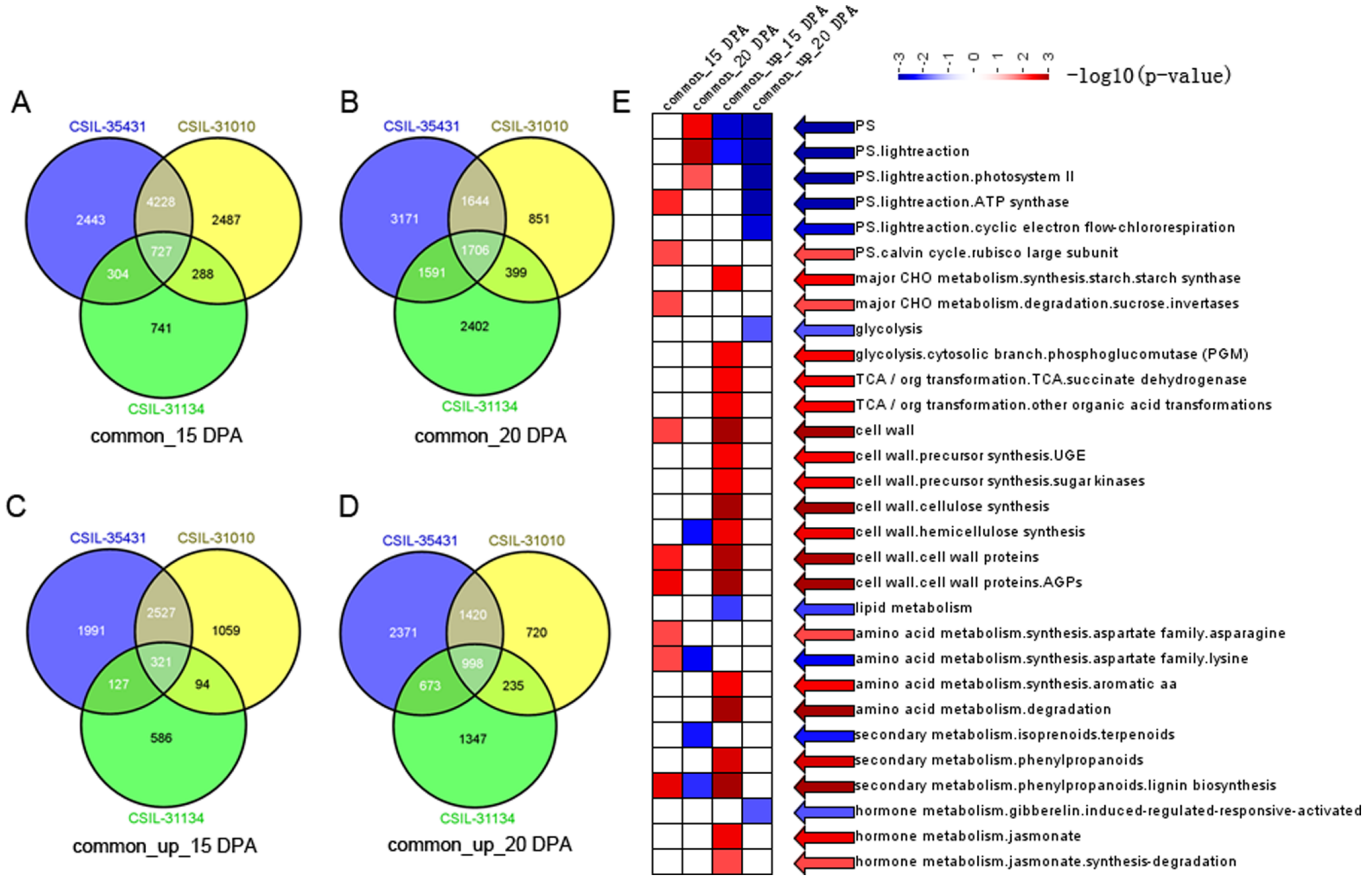
Analysis of common and common upregulated DEGs among three stronger fiber CSILs. (A, B, C, D) Common and common upregulated DEGs among three stronger fiber CSILs at 15 and 20 DPA. Common_up, common regulated DEGs. (B) Functional enrichment analysis of these DEGs using mapman software (Summary statistic type, wlcoxon). Colors from blue to red indicated that functions were enriched more significantly with smaller p-values.

To understand the mechanisms behind the changes in fiber strength observed in the CSILs, we also analyzed the common DEGs among CSIL-35431, CSIL-31010 and CSIL-31134 (Table S4). A total of 727 and 1796 common DEGs were selected at 15 and 20 DPA in three stronger fiber CSILs, respectively (Figure 3). More functional enrichment were shown at 15 DPA, including major CHO metabolism (carbohydrate), cell wall biosynthesis, amino acid metabolism and secondary metabolism (Figure 3E). Among these genes, 321 and 998 common upregulated DEGs between the same CSILs at 15 and 20 DPA were indentified, respectively (Figure 3). These common DEGs or processes maybe directly related to the fiber strength. However, these DEGs maybe function as downstream genes altered by the introgressed segments since these CSILs were inserted different *G. barbadense* segments in recipient TM-1.

**Figure 4:**
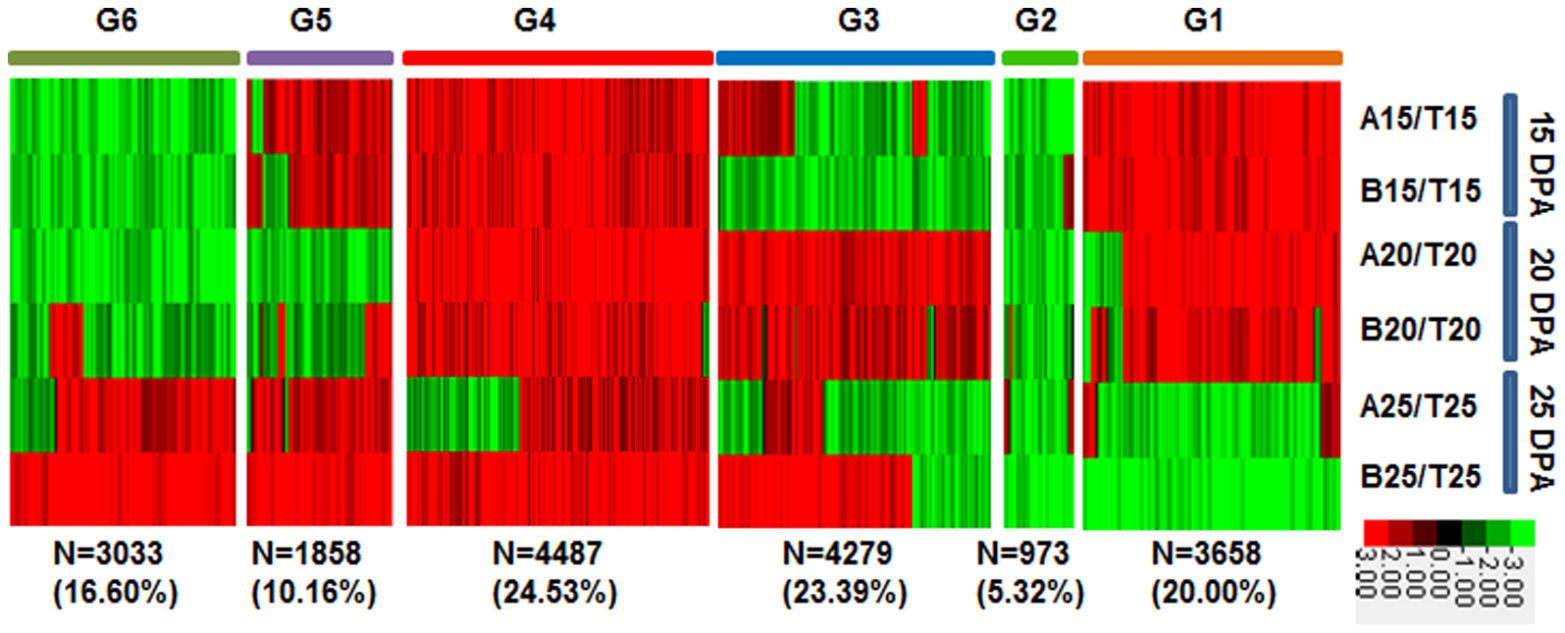
Heat map analysis of the expression of DEGs between CSILs and TM-1. A, B and T indicated CSIL-35431, CSIL-31010 and TM-1, respectively. 15, 15DPA; 20, 20DPA; 25, 25DPA. Red color indicated up-regulated genes and green color indicated down-regulated genes. N = number of DEGs in different group.

To visualize the expression patterns of DEGs, we performed cluster analysis of 18,288 genes that were differentally expressed between CSIL-35431 and CSIL-31010 (Figure 4). These DEGs could be grouped into six clusters, designated G1–G6, based on their expression patterns. From 15 DPA to 20 DPA, the stages of fast fiber elongation and secondary cell wall deposition overlap, with the latter reaching a peak at around 20–25 DPA. We focused on clusters G1, G4, and G6 to conduct data analysis in order to identify genes that were either up-regulated or down-regulated during the secondary cell wall synthesis stage. Compared to the TM-1 control, 3,658 genes in cluster G1 were highly expressed at 15 and 20 DPA, 4,487 genes in G4 were highly expressed at 15 DPA, 20 DPA, and 25 DPA, 3,033 genes in G6 were highly expressed only at 25 DPA, and the other three groups showed various different expression patterns. Clustering results for 19,742 DEGs from the four CSILs showed five groups, indicating that the gene expression pattern in CSIL-31134 was distinct from the others at 15 DPA and 20 DPA, and that CSIL-35368 was similar to CSIL35431 and CSIL-31010 (Figure S1).

### Functional annotation by GO enrichment and KEGG analysis

To understand the mechanisms behind the changes in fiber strength observed in the CSILs, we analyzed DEG enrichment in the major functional GO categories of biological process, molecular function, and cellular component between CSIL-35431 and CSIL-31010. Based on the clustering results shown in Figure 4, G1 was enriched in genes for secondary cell wall biogenesis, glucuronoxylan biosynthesis, microtubule-based movement, and cellulose biosynthesis, G4 was enriched in genes for protein phosphorylation, response to chitin, and sugar-mediated signaling pathways, and G6 was enriched in fatty acid biosynthesis genes (Table 2). These data suggest that in the developmental stage of secondary cell wall deposition, DEGs were enriched for carbohydrate synthesis and cell wall formation.

**Table 2:** Enrichment analysis of gene ontologies from 15 to 25 DPA.

Cluster	GO-ID	GO Ontology (Biological process)
G1	GO:0010417	glucuronoxylan biosynthetic process
15DPA up-regulated	GO:0009834	secondary cell wall biogenesis
20DPA up-regulated	GO:0007018	microtubule-based movement
25DPA down-regulated	GO:0030244	cellulose biosynthetic process
	GO:0009753	response to jasmonic acid stimulus
G2	GO:0015031	protein transport
15DPA down-regulated	GO:0015991	ATP hydrolysis
20DPA down-regulated	GO:0032544	plastid translation
25DPA down-regulated	GO:0016075	rRNA catabolic process
	GO:0006511	ubiquitin catabolic process
G3	GO:0009734	auxin mediated signaling pathway
15DPA down-regulated	GO:0030259	lipid glycosylation
20DPA up-regulated	GO:0018106	peptidyl-histidine phosphorylation
25DPA down-regulated	GO:0009722	detection of cytokinin stimulus
G4	GO:0006468	protein phosphorylation
15DPA up-regulated	GO:0010200	response to chitin
20DPA up-regulated	GO:0006096	glycolysis
25DPA up-regulated	GO:0010182	sugar mediated signaling pathway
	GO:0009966	regulation of signal transduction
G5	GO:0015884	protein folding
15DPA up-regulated	GO:0006886	intracellular protein transport
20DPA down-regulated	GO:0006122	mitochondrial electron transport
25DPA up-regulated	GO:0015914	phospholipid transport
G6	GO:0007267	cell-cell signaling
15DPA down-regulated	GO:0010025	wax biosynthetic process
20DPA down-regulated	GO:0006633	fatty acid biosynthetic process
25DPA up-regulated	GO:0006723	hydrocarbon biosynthetic process

G1–G6 according to Figure 3.

We applied the same GO analysis to the common DEGs at 15 DPA and 20 DPA in CSIL-35431 and CSIL-31010, respectively. These DEGs were enriched in genes for similar functional categories, such as cellular metabolic processes and carbohydrate metabolism, etc. We also found genes for some processes that were enriched only in CSIL35431 or CSIL-31010 (Figure S2).

Further GO analysis for CSIL-35368 and CSIL-31134 indicated that the DEGs in CSIL-35368 at 15 and 20 DPA were enriched in genes for lignin biosynthesis, secondary cell wall biogenesis, and response to chitin, which was similar to the enrichment found in CSIL-35431 and CSIL-31010. But at 15 and 20 DPA in the stronger fiber line CSIL-31134, GO enrichments were different from the other three lines, mainly in genes for ATP synthesis, proton transport, copper ion export, and oxidoreductase activity, but not in cell wall biosynthesis (Table S5).

Based on the results of GO analysis, we know that the secondary cell wall related biological process were impacted in the CSILs, but it is still not very clear how secondary cell wall biosynthesis was affected in the CSILs. Therefore, we performed pathway analysis on 18,288 DEGs in CSIL-35431 and CSIL-31010. The most highly enriched pathways found are listed in Table 3. KEGG analysis showed that the genes were enriched in pathways for starch and sucrose metabolism (328 genes), glycolysis/gluconeogenesis (122 genes), phenylpropanoid biosynthesis (101 genes), and oxidative phosphorylation (87 genes) (Table 3 and Figure S3). The regulation of some enzymes that catalyze sucrose, starch, and cellulose biosynthesis may have a direct or indirect impact on fiber quality. This could be especially true for sucrose and pectin metabolism, and many genes in these pathways were up-regulated. We also found that genes involved in phenylpropanoid and flavonoid biosynthetic processes were enriched in the CSILs.

**Table 3:** KEGG analysis of DEGs in CSIL-35431 and CSIL-31010.

Pathway	DEGs with pathway annotation (2576)	References genes with pathway annotation(4601)	Ratio	DEGs distribution in each groups					
				G1	G2	G3	G4	G5	G6
Starch and sucrose metabolism	328	563	58.26%	87	15	54	89	31	52
Purine metabolism	251	459	54.68%	35	20	53	65	30	48
Phenylalanine metabolism	140	261	53.64%	29	5	26	47	12	21
Amino sugar and nucleotide sugar metabolism	137	211	64.93%	47	5	23	36	12	14
Pyrimidine metabolism	128	227	56.39%	15	13	26	37	15	22
Glycolysis/Gluconeogenesis	122	188	64.89%	28	4	23	35	13	19
T cell receptor signaling pathway	115	212	54.25%	28	4	23	34	11	15
Pentose and glucuronate interconversions	109	227	48.02%	24	8	20	27	7	23
Glycerolipid metabolism	102	174	58.62%	18	4	29	21	15	15
Pyruvate metabolism	102	182	56.04%	16	8	18	29	12	19
Phenylpropanoid biosynthesis	101	220	45.91%	26	6	17	28	8	16
Galactose metabolism	94	160	58.75%	23	2	20	24	7	18
Cysteine and methionine metabolism	94	145	64.83%	21	2	21	28	10	12
Glycerophospholipid metabolism	94	167	56.29%	14	8	29	21	10	12
Arginine and proline metabolism	93	144	64.58%	16	5	22	19	12	19
Oxidative phosphorylation	87	184	47.28%	19	13	12	11	13	19
Carbon fixation in photosynthetic organisms	84	151	55.63%	19	3	11	34	8	9
Fatty acid degradation	83	133	62.41%	17	5	18	21	7	15

G1–G6 according to Figure 3.

Based on the cluster analysis of the weaker fiber line CSIL-35368, we hypothesized that changes in other biochemical pathways led to reduced fiber strength (Figure S1). Considering only those that were down-regulated in CSIL-35368, we found genes that participated in fatty acid degradation and biosynthesis, and also in carbohydrate metabolic pathways (Figure 2B and Figure S4).

**Figure 5:**
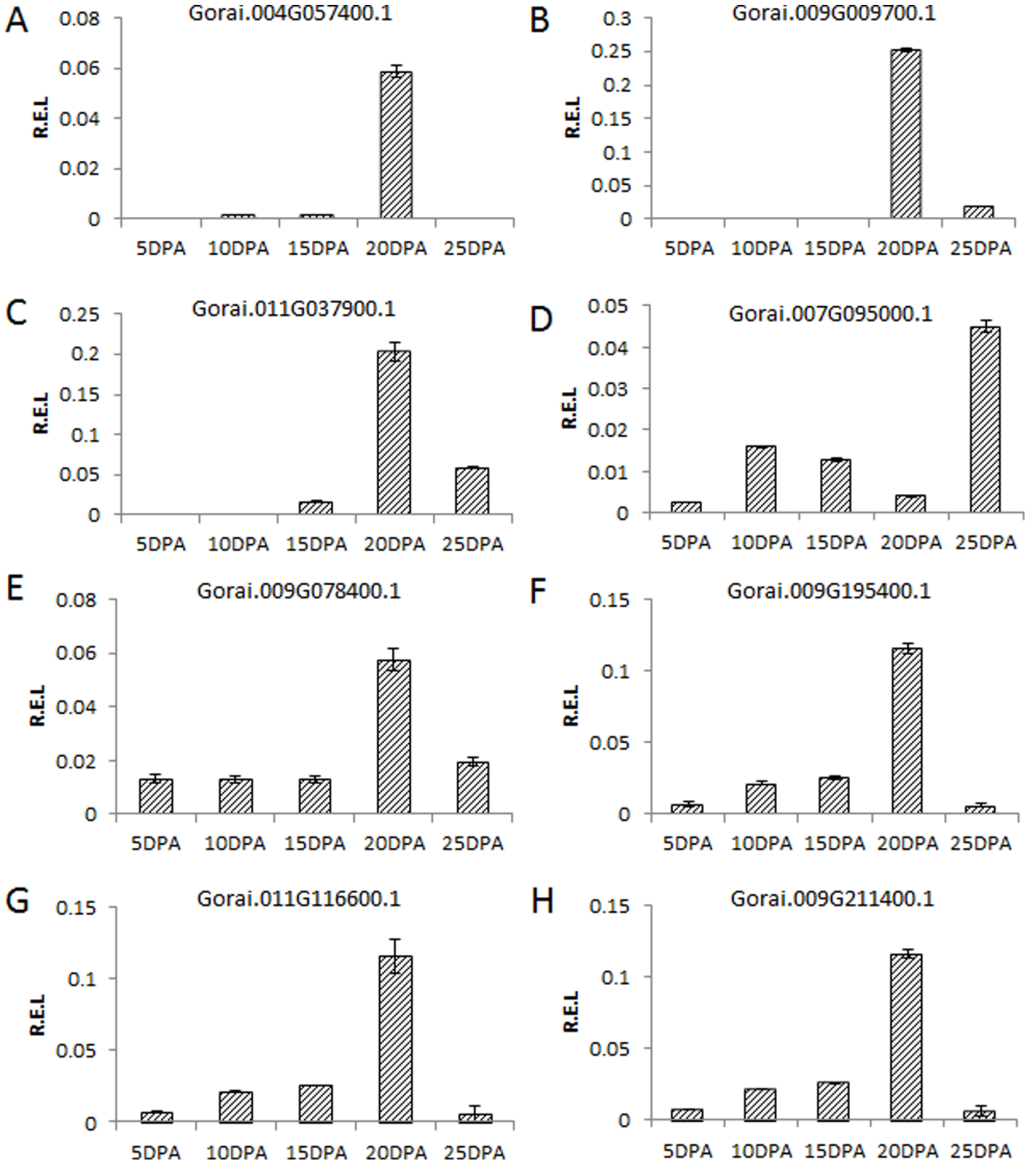
Quantitative RT–PCR validation of tag-mapped genes in TM-1. These genes have been reported before, including 3 CesA genes (A,B,C) (homologous with AtCESA4, AtCESA7, AtCESA8, respectively), xyloglucan endotransglucosylase (D), beta -galactosidase (E), glycosyl hydrolase 9B7 (F), xylan alpha-glucuronosyltransferase 1, GUX1 (G), xylan alpha-glucuronosyltransferase 2, GUX2 (H).

**Figure 6:**
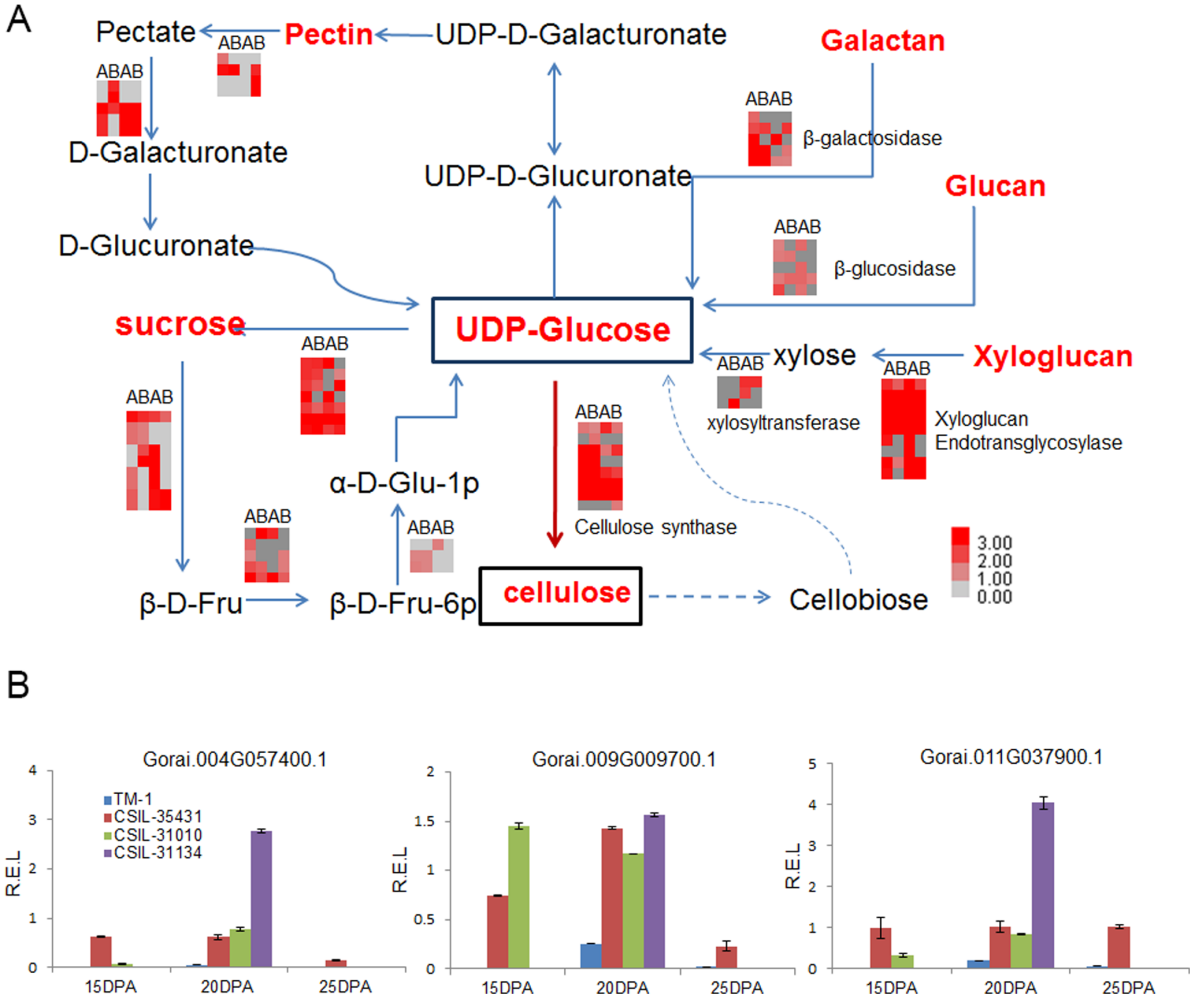
Carbohydrate pathways that are differentially regulated during the secondary cell wall synthesis stage. (A) Carbohydrate pathways. Genes up-regulated in CSIL-315431 and CSIL-31010 were selected to do heat map. ABAB indicated DEGs in CSIL-35431 at 15 DPA, CSIL-35431 at 20DPA, CSIL-3010 at 15DPA and CSIL-31010 at 20DPA, from left to right. Every square stand for one gene and every line stand for the same gene. Genes with red color expressed higher in CSILs than TM-1 and gray color stand for no difference. β-D-Fru, β-D-Fructose; α-D-Glu-1p, α-D-Glucose-1-phosphate; β-D-Fru-6p, β-D-Fructose-6-phosphate. (B) Quantitative RT–PCR validation of four CesA genes in CSILs and TM-1, Gorai.004G057400.1, Gorai.009G009700.1 and Gorai.011G037900.1 homologous with *AtCESA4*, *AtCESA7* and *AtCESA8*, respectively.

**Figure 7:**
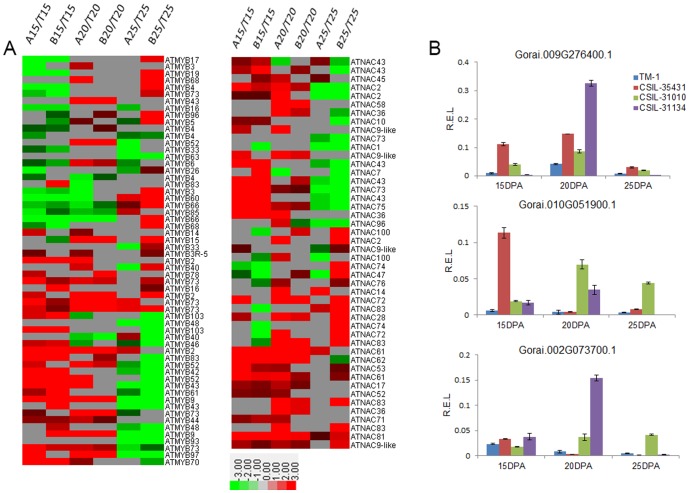
NAC and MYB family genes involved in the regulation of secondary wall biosynthesis. (A) 59 MYB family genes and 47 NAC family genes showed different expression level between CSILs and TM-1 at 15DPA, 20DPA and 25DPA. |Ratio|>2 and FDR<0.001. A, B, T indicated CSIL-35431, CSIL-31010 and TM-1. 15, 15DPA; 20, 20DPA; 25, 25DPA. (B) Quantitative RT–PCR validation of three transcription factors.

Eight genes previously reported in the carbohydrate pathway were selected for quantitative RT-PCR. The expression patterns of these genes were consistent with the DEG data in TM-1 (Figure 5) and in the CSILs as well (Figure 6B and Figure 7B).

### Carbohydrate metabolism in the secondary cell wall synthesis stage

Following the start of secondary cell wall formation, protein and carbohydrate metabolism genes involved in cell wall biosynthesis will be up-regulated [27]. We selected 72 DEGs associated with carbohydrate metabolism to investigate the mechanism of fiber development. These genes were related to pectin, sucrose, galactan, glucan, xyloglucan, and cellulose biosynthesis. We were interested in genes that are up-regulated in fiber cells at 15 DPA and 20 DPA, at the start of secondary cell wall formation. A heat map showing the different expression levels for these genes including cellulose synthase, sucrose synthase, pectin lyase, and other polysaccharides degradation in CSIL-35431 and CSIL-31010 is shown in Figure 6A. We found that the cellulose synthase genes were up-regulated in the CSILs at 15 DPA-25 DPA. It has been reported that cellulose biosynthesis predominates, and that many other metabolic pathways are down-regulated during secondary cell wall synthesis [27]. Moreover, we confirmed the expression patterns of cellulose synthase genes, annotated with the *Arabidopsis* genes *AtCESA4*, *AtCESA7* and *AtCESA8*, using quantitative RT-PCR (Figure 4B). Proteins encoded by *AtCESA4*, *7*, and *8* are specifically required to form a functional cellulose synthase complex (CSC) that is essential for secondary cell wall formation [40]–[42].

### Transcription factors associated with secondary cell wall synthesis

Recent molecular and genetic studies have identified transcription factors that are involved in regulating secondary cell wall synthesis in *Arabidopsis* [43]–[45]. In our study, 97 MYB-type and 68 NAC-type transcription factors showed changes in expression between the CSILs and TM-1 (Table S6, Table S7). It was interesting that some NACs and MYBs were up-regulated in CSIL-35431 and CSIL-31010 during the secondary cell wall synthesis stage, especially at 15 DPA and 20 DPA. Defined as |log_2_Ratio|≥2, 59 MYB and 47 NAC transcription factors were selected for heat-map analysis (Figure 7A). Among these transcription factors, genes homologous with *ATMYB2*, *ATMYB43*, *ATMYB73*, *ATNAC52*, and *ATNAC61* were expressed at higher levels in the CSILs. We confirmed that three transcription factors were up-regulated in CSILs from 15 DPA to 25 DPA (Figure 7B). In the MYB family, it has been reported that the expression of genes for MYB85, MYB52, MYB54, MYB69, MYB42, and MYB43 are developmentally associated with cells undergoing secondary wall thickening [45].

### Different metabolic pathways associated with altered fiber strength

**Figure 8:**
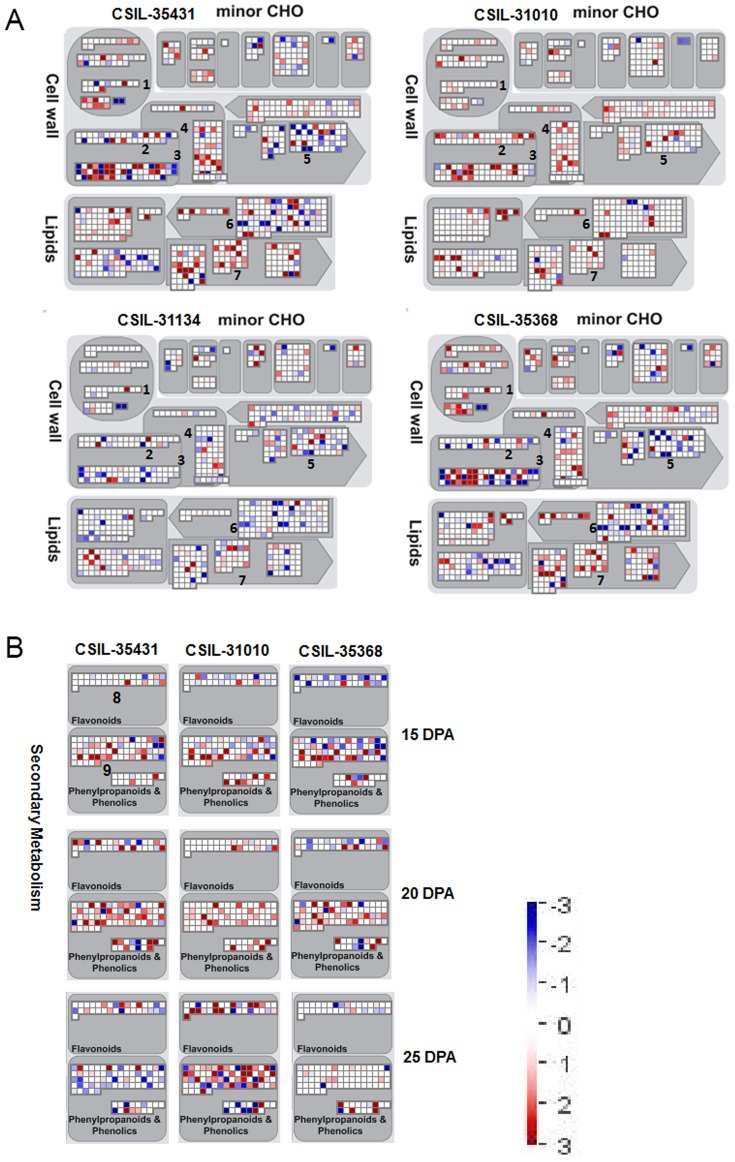
Metabolism analysis of DEGs in CSILs during the secondary cell wall biosynthesis stage. (A) Motabolism overview in four CSILs at 20 DPA. (B) Secondary motabolism analysis in three CSILs at 15 DPA, 20 DPA and 25 DPA. 1, cell wall protein; 2, cell wall pectin esterases; 3, cell wall modification; 4, cell wall cellulose synthesis; 5, cell wall degradation/pectate lyases; 6, lipid metabolism/FA synthesis; 7, lipid degradation; 8, flavonoids; 9, phenylpropanoids/lignin biosynthesis. Blue square, down-regulated genes; Red square, up-regulated genes.

In order to investigate the mechanisms underlying changes in fiber strength, we analyzed several metabolic pathways including cell wall, lipids, minor CHO (carbohydrate) metabolism, and two secondary metabolite pathways. It is interesting that DEGs involved in cell wall proteins, cell wall pectin esterase, cell wall modification, cell wall cellulose synthesis, cell wall degradation/pectate lyases, lipid metabolism/FA synthesis, and lipid degradation showed distinct expression patterns or differential up/down-regulation at 20 DPA (Figure 8A). We found that up-regulated DEGs were similar to down-regulated DEGs both in CSIL-35431 and CSIL-35368. However, most of DEGs in CSIL-31010 were up-regulated at 20 DPA, while the opposite was true for DEGs in CSIL-31134, especially those genes involved in cell wall modification. In CSIL-31134, we also found a few genes in these metabolic pathways that were changed at 15 DPA except in cell wall modification, and in CSIL-31010, we found DEGs enriched in these metabolic pathways at 25 DPA (Figure S5). From the secondary metabolism results, we identified a few DEGs involved in flavonoid biosynthesis in CSIL-35431 and CSIL-31010 at 15 DPA. In contrast, more genes were up-regulated or down-regulated in CSIL-35368 at 15 DPA. It was obvious that DEGs from the phenylpropanoid pathways at 25 DPA were different from one another, and the expression pattern of DEGs in CSIL-31010 changed dramatically. Moreover, there were few genes that were up-regulated or down-regulated in CSIL-35368 at 25 DPA (Figure 7B). We assume that metabolic pathways in the CSILs at different developmental stages were changed in various ways as a result of the introgressed chromosmal segments from *G. barbadense*.

## Discussion

*G. hirsutum* produces a high yield of cotton with moderate fiber strength. *G. barbadense* is characterized by a low yield, but with increased fiber fineness and strength. As a breeding target, we tried to combine the high yield of *G. hirsutum* with the superior fiber qualities of *G. barbadense*, and we also wanted to elucidate the molecular mechanism behind the formation of superior quality fibers. Fiber strength is an important indicator of the cotton fiber quality, and depends on the formation of the secondary cell wall. Genome-wide transcriptome profiling is effective at revealing significant genes and pathways involved in secondary cell wall formation. Transcriptome analysis showed that gene expression patterns and functional distribution were different during secondary cell wall biosynthesis.

### Carbohydrate metabolism plays an important role in secondary cell wall synthesis

It is well known that the mature cotton fiber is composed of nearly pure cellulose, and that such a high level of cellulose synthesis requires an abundant supply of UDP-glucose [46], [47]. This means that a large amount of cellulose is required during the secondary cell wall synthesis stage. Functional classification and enrichment analysis showed that following the initiation of secondary cell wall synthesis, DEGs were enriched for secondary cell wall biogenesis, glucuronoxylan biological processes, and other carbohydrate metabolic pathways in the CSILs (Table 2). Focusing on carbohydrate metabolic pathways, it is obvious that the key intermediate in the multiple pathways is UDP-glucose, a substrate for cellulose synthesis. Our results showed that several CesA genes are expressed at higher levels during secondary cell wall synthesis than they are at earlier stages (Figure 6B). Ten *AtCESA* genes have been reported in *Arabidopsis*, and *AtCESA4*, *7*, and *8* are specifically required to form the cellulose synthase complex (CSC) that is essential for secondary cell wall synthesis [40]–[42]. Similarly, three CESA isoforms have been identified during secondary cell wall synthesis in rice, maize, and *Populus* [10], [48], [49]. Also, many genes that participate in the degradation of poly- and oligo-saccharides were found to be up-regulated at 15 and 20 DPA, in order to produce more UDP-glucose for cellulose biosynthesis. Similarly, it has also been reported that during the secondary cell wall synthesis stage, certain metabolic pathways, including hydrolysis of fatty acids and non-cellulose poly- and oligo-saccharides, would be up-regulated [27]. Sucrose synthase (SuSy) has long been studied as a cytoplasmic enzyme in plant cells, where it serves to degrade sucrose and provide carbon for respiration and synthesis of cell wall polysaccharides and starch [50]. It has also been shown that genes associated with secondary cell wall biosynthesis are involved in sugar metabolism [51].

### Multiple mechanisms affect fiber strength development

Except for carbohydrate metabolism, recent research has shown that transcription factors also affect fiber development during secondary cell wall biosynthesis. Several NAC- and MYB-type transcription factors were up-regulated in the CSILs compared to TM-1 from 15 DPA to 25 DPA, and these included cotton homologs of *AtMYB2*, *AtMYB43*, and *AtNAC52* etc. (Figure 7A). The NAC-mediated transcriptional regulation of secondary wall biosynthesis is a conserved mechanism throughout vascular plants [44], [52]. *SND2*, a NAC transcription factor gene, regulates genes involved in secondary cell wall development in *Arabidopsis* fibers and increases fiber cell area in *Eucalyptus* [53]. A MYB75-associated protein complex is likely to be involved in modulating secondary cell wall biosynthesis in both the *Arabidopsis* inflorescence and stem [54]. It has also been found that the rice and maize MYB transcription factors, OsMYB46 and ZmMYB46, are functional orthologs of *Arabidopsis* MYB46/MYB83 and, when overexpressed in *Arabidopsis*, are able to activate the entire secondary wall biosynthetic program [55].

Several metabolic pathways were examined to determine the mechanism behind changes in fiber strength; these included cell wall, lipids, minor CHO metabolism, and two secondary metabolic pathways. Although results of the GO and KEGG analyses showed that CSIL-35431, CSIL-31010, and CSIL-35368 had similar patterns, fiber strength in these three lines were different. Our results support the hypothesis that different metabolic pathways can affect fiber strength, and the same pathway in the CSILs can be altered differentially at various times in development. DEGs in CSIL-31010 were up-regulated at 20 DPA, while the opposite was found for DEGs in CSIL-31134, especially those genes involved in cell wall modification. The expression levels of genes involved in flavonoid biosynthesis in the weak fiber line CSIL-35368 were changed dramatically at 15 DPA, but there were few genes changed at 25 DPA; this patter was the opposite of that in CSIL-35431 and CSIL-31010, lines with high quality fiber. We hypothesize that phenylpropanoid and flavonoid metabolism generally affected the fiber strength of CSIL-35368. Genes for phenylpropanoid and flavonoid biosynthesis showed significant enrichment and temporal differences in gene expression patterns which are associated with xylem formation [56]. It has been reported that expression levels of phenylpropanoid genes showed high correlations with specific fiber properties, supporting a role in determining fiber strength [57].

In conclusion, upland cotton CSILs carrying distinct *G. barbadense* chromosomal segments provide valuable material for research into fiber development. The *G. barbadense* chromosome segments resulted in different patterns of differentially expressed genes, and altered different metabolic pathways, mainly in carbohydrate metabolism. In addition, several transcription factor genes were found to be specifically up-regulated in the CSILs. Metabolic pathways involved in cell wall, lipid, phenylpropanoid, and flavonoid biosynthesis play a significant role during secondary cell wall formation, and are associated with the development of cotton fiber strength.

## Supporting Information

Figure S1 **Heat map of the expression of DEGs between 4 CSILs at 15–25 DPA.**
(TIF)

Figure S2 **Enrichment analysis of common DEGs at 15DPA and 20DPA in CSIL-35431 and CSIL-31010.**
(TIF)

Figure S3 **Heat map of DEGs participated in four metabolic pathways from 15 DPA to 25 DPA.**
(TIF)

Figure S4 **Pathway analysis of genes only down-regulated in CSIL-35368 from 15 DPA to 25 DPA.**
(TIF)

Figure S5 **Metabolism analysis of DEGs in CSILs at 15 DPA and 25 DPA.**
(TIF)

Table S1 **Average fiber quality of 4 CSILs and TM-1.**
(XLS)

Table S2 **Primer for quantitative RT-PCR.**
(XLS)

Table S3 **Categorization and abundance of tags.**
(XLS)

Table S4 **List of common DEGs among CSIL-35431, CSIL-31134 and CSIL-31010.**
(XLS)

Table S5 **Enrichment analysis of gene ontologies in CSIL-35368 and CSIL-31010 at 15 DPA and 20 DPA.**
(XLS)

Table S6 **Different expression level of 97 MYB transcription factors.**
(XLS)

Table S7 **Different expression level of 68 NAC transcription factors.**
(XLS)
