# Supplementary figures and images for: Transcriptomic Analysis of Fiber Strength in Upland Cotton Chromosome Introgression Lines Carrying Different Gossypium barbadense Chromosomal Segments
Source: PLoS One. 2014 Apr 24;9(4):e94642. doi: 10.1371/journal.pone.0094642 (PMC3998979; doi:10.1371/journal.pone.0094642)

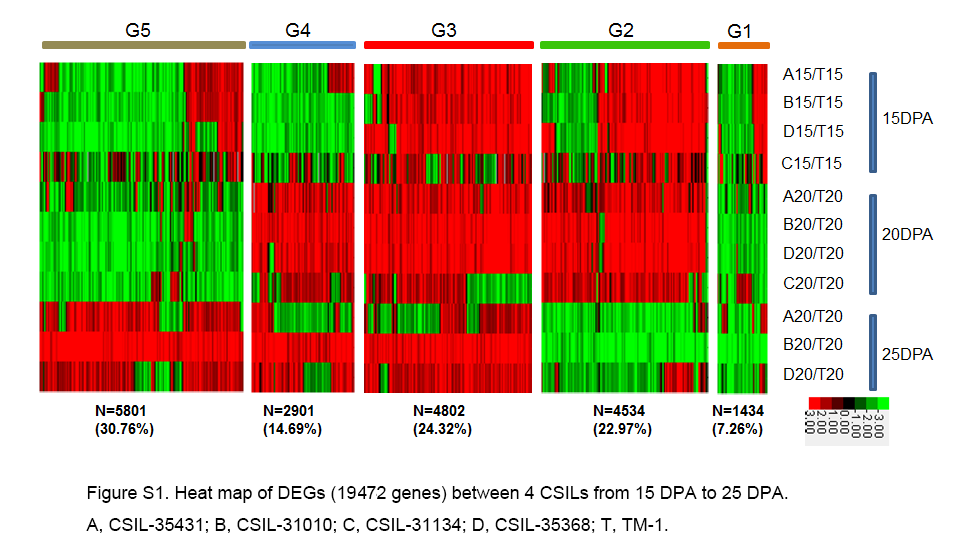

Supplement: Figure S1 — Heat map of the expression of DEGs between 4 CSILs at 15–25 DPA. (TIF) [file pone.0094642.s001.tif]

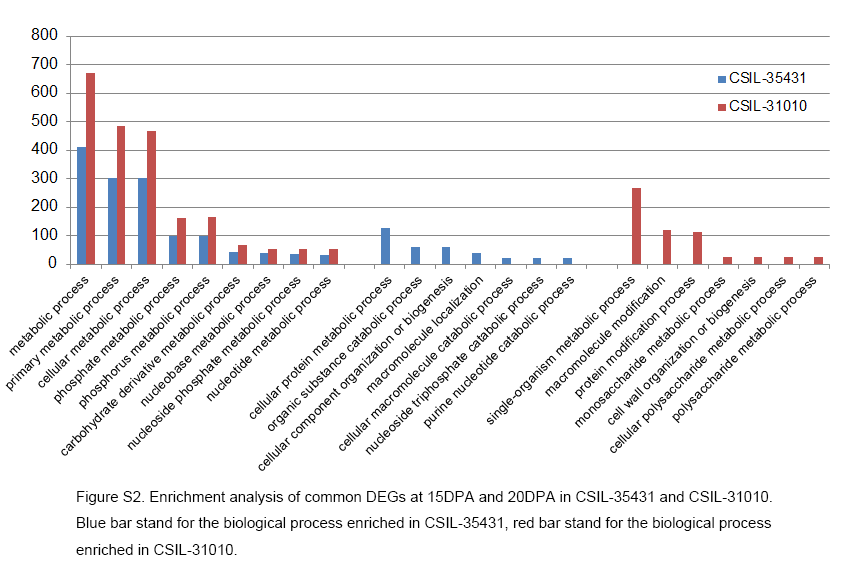

Supplement: Figure S2 — Enrichment analysis of common DEGs at 15DPA and 20DPA in CSIL-35431 and CSIL-31010. (TIF) [file pone.0094642.s002.tif]

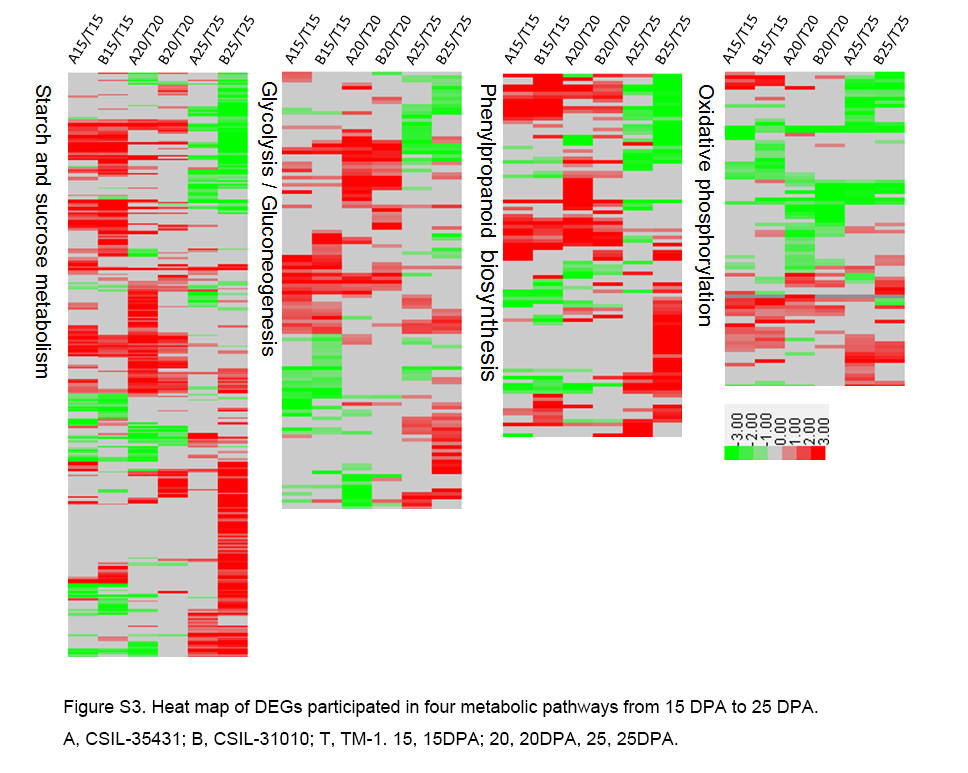

Supplement: Figure S3 — Heat map of DEGs participated in four metabolic pathways from 15 DPA to 25 DPA. (TIF) [file pone.0094642.s003.tif]

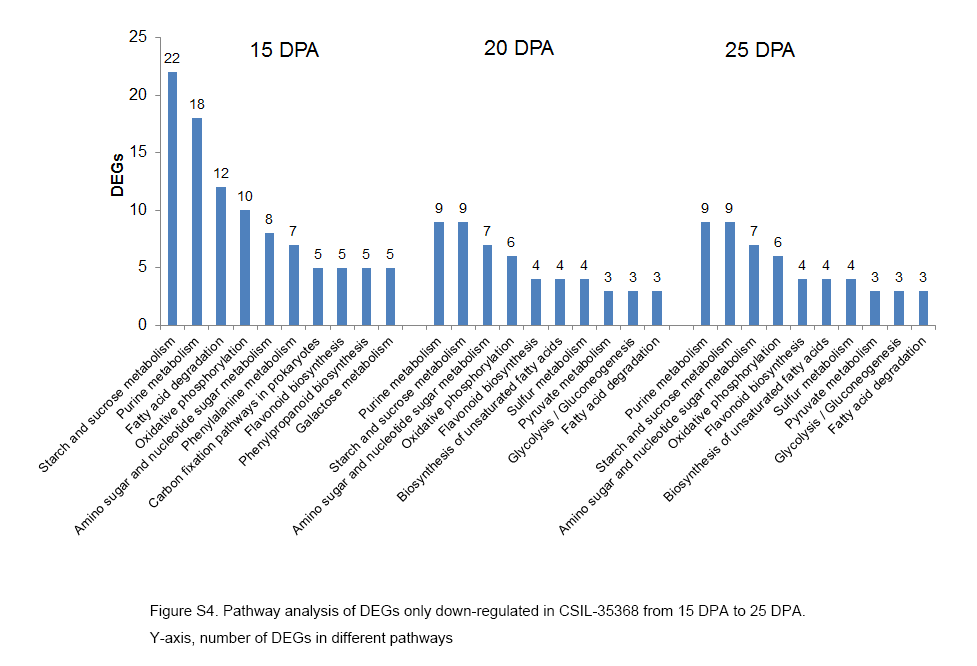

Supplement: Figure S4 — Pathway analysis of genes only down-regulated in CSIL-35368 from 15 DPA to 25 DPA. (TIF) [file pone.0094642.s004.tif]

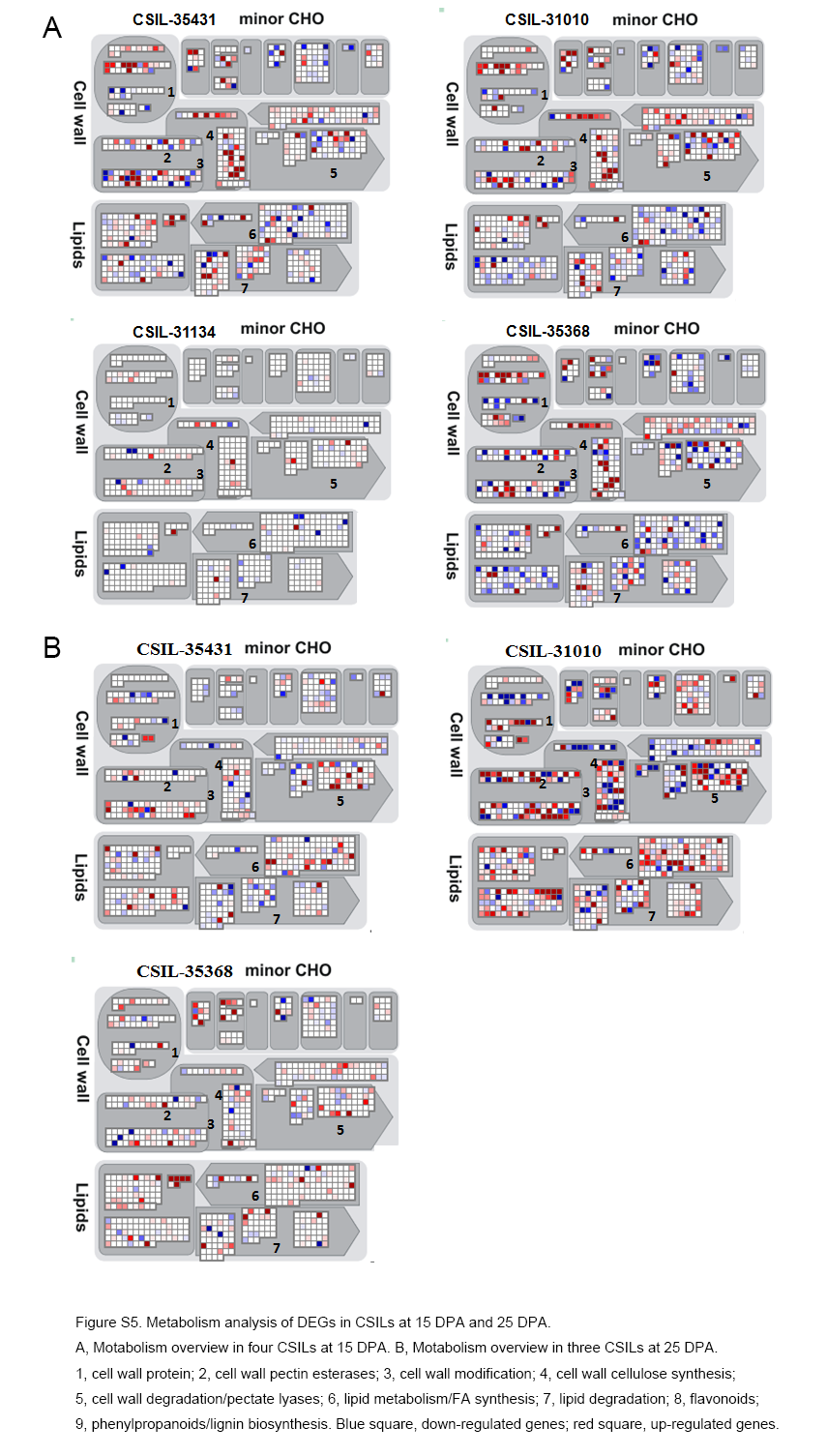

Supplement: Figure S5 — Metabolism analysis of DEGs in CSILs at 15 DPA and 25 DPA. (TIF) [file pone.0094642.s005.tif]
